# Dieckol Decreases Caloric Intake and Attenuates Nonalcoholic Fatty Liver Disease and Hepatic Lymphatic Vessel Dysfunction in High-Fat-Diet-Fed Mice

**DOI:** 10.3390/md19090495

**Published:** 2021-08-30

**Authors:** Kyung-A Byun, Seyeon Oh, Myeongjoo Son, Chul-Hyun Park, Kuk Hui Son, Kyunghee Byun

**Affiliations:** 1 Department of Anatomy & Cell Biology, College of Medicine, Gachon University, Incheon 21936, Korea; kabyun95@gmail.com (K.-A.B.); mjson@gachon.ac.kr (M.S.); 2 Functional Cellular Networks Laboratory, Lee Gil Ya Cancer and Diabetes Institute, College of Medicine, Gachon University, Incheon 21999, Korea; seyeon8965@gmail.com; 3 Department of Thoracic and Cardiovascular Surgery, Gil Medical Center, Gachon University, Incheon 21565, Korea; cdgpch@gilhospital.com

**Keywords:** nonalcoholic fatty liver disease, *Ecklonia cava* extracts, dieckol, lymphangiogenesis, lymphatic permeability

## Abstract

Increased inflammation is the main pathophysiology of nonalcoholic fatty liver disease (NAFLD). Inflammation affects lymphatic vessel function that contributes to the removal of immune cells or macromolecules. Dysfunctional lymphatic vessels with decreased permeability are present in NAFLD. High-fat diet (HFD) is known to increase body weight, food intake, and inflammation in the liver. Previously, it was reported that *Ecklonia cava* extracts (ECE) decreased food intake or weight gain, and low-calorie diet and weight loss is known as a treatment for NAFLD. In this study, the effects of ECE and dieckol (DK)—which is one component of ECE that decreases inflammation and increases lymphangiogenesis and lymphatic drainage by controlling lymphatic permeability in high-fat diet (HFD)-fed mice—on weight gain and food intake were investigated. ECE and DK decreased weight gain and food intake in the HFD-fed mice. NAFLD activities such as steatosis, lobular inflammation, and ballooning were increased by HFD and attenuated by ECE and DK. The expression of inflammatory cytokines such as IL-6 and TNF-α and infiltration of M1 macrophages were increased by HFD, and they were decreased by ECE or DK. The signaling pathways of lymphangiogenesis, VEGFR-3, PI3K/pAKT, and pERK were decreased by HFD, and they were restored by either ECE or DK. The expression of VE-cadherin (which represents lymphatic junctional function) was increased by HFD, although it was restored by either ECE or DK. In conclusion, ECE and DK attenuated NAFLD by decreasing weight gain and food intake, decreasing inflammation, and increasing lymphangiogenesis, as well as modulating lymphatic vessel permeability.

## 1. Introduction

Nonalcoholic fatty liver disease (NAFLD) is one of the major chronic metabolic disorders that lead to increased all-cause mortality [1]. NAFLD encompasses a broad spectrum of diseases, from nonalcoholic fatty liver (NAFL) or isolated hepatic steatosis to nonalcoholic steatohepatitis (NASH) [2,3]. The histologic findings of isolated hepatic steatosis are excessive fat accumulation without injury or inflammation, whereas NASH showed hepatocyte ballooning, liver injury, inflammation, and fibrosis, which lead to cirrhosis [4,5]. Excessive deposition of fat in the liver parenchyma results from the imbalance between the excessive intake of high-fat diet (HFD) or increased newly synthesized lipid by de novo lipogenesis and decreased lipid clearance, predominantly through free fatty acid (FFA) oxidation [6]. Many studies have demonstrated that HFD induces NAFLD in animals and overconsumption of HFD is associated with NAFLD in humans [7,8].

The development and progression of NAFLD are associated with inflammation. Pure steatosis is not involved in the disease progression of NAFLD, but fibrosis has been shown to accelerate NAFLD to a more severe grade, leading to mortality [9]. In hepatocytes, extreme FFAs are one of the major inducers of inflammation. Many studies have shown the association between obesity, which increases inflammation, and liver disease [9,10]. Various proinflammatory cytokines or inflammatory cytokines were increased in the blood circulation by HFD [9]. In fatty liver disease, hepatic tumor necrosis factor (TNF)-α, interleukin (IL)-6, and IL-1β are increased, and these are more increased by the aggravation of NAFLD to more severe stages [10].

Kupffer cells and macrophages recruited into the liver are involved in the development of NASH by increasing the activation of the macrophage M1 phenotype and decreasing that of the M2 phenotype [11,12]. M1 macrophages secrete various cytokines, such as IL-1β, IL-12, and TNF-α, which recruit proinflammatory immune cells and aggravate local inflammation [13,14].

The lymphatic vessels in the liver have a role in draining interstitial fluid, fat, cholesterol, and immune cells [15]. Lymphatic vessels are covered by a single layer of lymphatic endothelial cells (LyECs), without smooth muscle cells, that lack a continuous basement membrane. Thus, those structures form highly permeable button-like junctions [16,17,18,19,20,21]. Those junctions allow the transport of interstitial fluid, macromolecules such as proteins or lipids, and immune cells [16,17,18,19,20,21]. After birth, lymphatic vessels are generally quiescent, but inflammation-related pathologic conditions, tissue repair, and tumor-related conditions induce lymphangiogenesis [22]. Vascular endothelial growth factor receptor (VEGF)-C/D and its receptor of VEGFR-3 are the most well-known signaling pathways that induce lymphangiogenesis [23]. Binding VEGF-C or VEGF-D to VEGFR-3 results in the autophosphorylation of VEGFR-3 [24] and upregulation of the Ras/Raf/MEK/ERK signaling pathway [25]. The autophosphorylation of VEGFR-3 also increases the PI3K/Akt pathway, which causes phosphorylation of Akt, leading to the upregulation of the mammalian target of rapamycin (mTOR) and Rac1 [26,27]. The activation of these signaling pathways induces the proliferation and migration of LyECs, leading to lymphangiogenesis [26]. It was suggested that increased lymphangiogenesis would be beneficial in resolving inflammation by removing infiltrated immune cells or inflammatory mediators from inflamed tissue [28,29,30].

Obesity and HFD are known to induce lymphatic dysfunction. Previous studies have demonstrated that the lymphatic density in the subcutaneous tissues of HFD-induced obese animals was decreased [31,32,33]. The decreased lymphatic density was associated with reduced LyEC proliferation, increased lymphatic leakage, decreasing pumping capacity of lymphatic vessels, and impairment in the removal of macromolecules [31,32,33]. The level of FFAs, which is increased in tissues of obese humans and animals, leads to inflammation in various tissues [34]. It is known that FFAs induce apoptosis or necrosis of blood endothelial cells [35]. FFAs also induce significant damage to LyECs even at low concentrations and result in increased apoptosis, decreased LyEC proliferation, and decreased expression of prospero-related homeobox 1 (*PROX-1*), which is a marker of LyECs [32]. FFAs also lead to decreased expression of VEGFR-3 [32]. In addition, impaired lymphatic vessels cause decreasing cholesterol clearance. In the skin of hypercholesterolemic mice, lymphatic dysfunction was observed, and the expression of VEGF-C was decreased [36]. The administration of VEGF-C improved lymphatic function and increased cholesterol clearance in those mice [36].

It was demonstrated that lymphatic vessel density was increased in the livers of NASH human subjects or NASH mice model induced by HFD [37]. Even though lymphatic vessel density was increased, the expressions of the lymphatic genes of LyECs such as podoplanin (*PDPN*), lymphatic vessel endothelial receptor-1 (*LYVE-1*), *PROX-1*, and *VEGFR-3* were decreased [37]. Those changes were associated with the dedifferentiation of LyECs, which induced changes in cell-to-cell junctions and decreased lymphatic drainage [37]. The expression of VE-cadherin in the LyECs of HFD-fed mice was higher than that in control-diet-fed mice [37]. VE-cadherin is an essential adhesion molecule that is involved in the formation of vascular structures and provides stability for vascular junctions [38]. It is known that decreased VE-cadherin leads to junctional disruption and excessive vessel permeability, resulting in lymphedema [39,40].

However, the overexpression of VE-cadherin was associated with decreasing ability to transport FITC-dextran through the LyEC junctions. Thus, the overexpression of VE-cadherin might represent impairments in lymphatic permeability that are associated with decreased lymphatic drainage [37]. The administration of recombinant VEGF-C decreased inflammation in the livers of HFD-induced NASH animal models by increasing lymphangiogenesis and restoring lymphatic function [37]. 

It was demonstrated that phlorotannins from *Ecklonia cava* extracts (ECE) decreased inflammation in white fat tissue by modulating M1/M2 macrophages [41]. ECE showed an attenuating effect on the inflammation of perivascular fat tissue by decreasing macrophage infiltration and decreasing proinflammatory cytokines such as TNF-α and IL-6 [42].

Dieckol (DK) is one of the phlorotannins from ECE (Figure S1). Phlorotannins are a class of compounds with polymerized phloroglucinol units [43]. DK has 11 OH groups in its structure [44]. DK from *Laminaria japonica* is known to decrease hepatic steatosis by increasing hepatic fatty acid β-oxidation [45]. However, it has not been revealed whether ECE or DK decreased NAFLD by increasing lymphangiogenesis and restoring impaired lymphatic vessel function in the liver. We hypothesized that ECE and DK decreased the infiltration of M1 macrophages and inflammatory cytokines such as IL-6 and TNF-α in the livers of HFD-fed mice. Decreased tissue inflammation might induce the upregulation of VEGFR-3, leading to lymphangiogenesis and the restoration of impaired lymphatic functions. Thus, we evaluated whether ECE or DK attenuated NAFLD by decreasing tissue inflammation and restoring lymphatic function in the livers of HFD-fed mice.

## 2. Results

### 2.1. ECE and DK Decreased Food Intake and Change of Bodyweight and Improved Serum Lipid Profile and NAFLD Activity of HFD-Fed Mice

**Figure 1:**
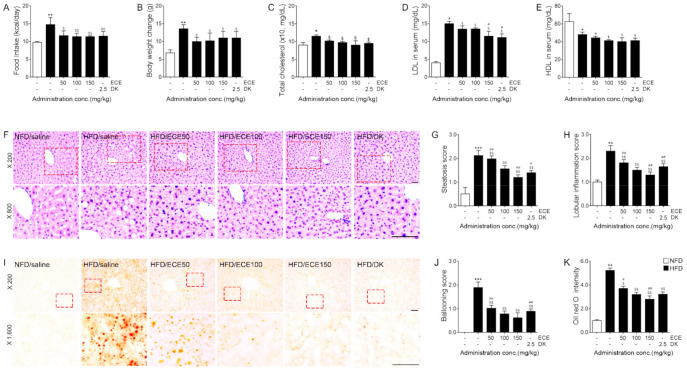
Effects of ECE and DK on the food intake, bodyweight change, serum lipid profile, and NAFLD activity in the livers of HFD-fed mice. (**A**,**B**) Food intake and change of bodyweight were increased by HFD/saline and decreased by ECE or DK treatment. (**C**,**D**) The serum levels of total cholesterol (**C**) and LDL (**D**) were increased by HFD/saline and decreased by ECE or DK treatment. (**E**) The serum level of HDL was decreased by HFD/saline, and decreased more by ECE or DK treatment. (**F**–**H**,**J**) The NAFLD activity was scored by hematoxylin and eosin staining (**F**). The hepatic steatosis score (**G**), lobular inflammation score (**H**), and ballooning score (**J**) were increased by HFD/saline and decreased after treatment with ECE or DK. (**I**,**K**) The hepatic lipid deposition by oil red O staining was increased by HFD/saline and decreased after treatment with ECE or DK. Scale bar = 100 μm. Data are mean ± SD. * *p* < 0.05, ** *p* < 0.01, and *** *p* < 0.001 vs. NFD/saline; $ *p* < 0.05 and $$ *p* < 0.01 vs. HFD/saline; # *p* < 0.05 and ## *p* < 0.01 vs. HFD/ECE100 (Mann–Whitney U test). DK, dieckol; ECE, *Ecklonia cava* extract; HFD, high-fat diet; HDL, high-density lipoprotein; LDL, low-density lipoprotein; NFD, normal fat diet.

The food intake of HFD-fed mice was significantly higher than that of NFD-fed mice (Figure 1A). The food intake was significantly decreased by 50, 100, and 150 mg/kg of ECE and DK. The change in bodyweights (time point between the starting day of HFD and the day of harvesting livers) of HFD-fed mice were significantly higher than those of NFD-fed mice. The change of bodyweight was significantly decreased by 50, 100, and 150 mg/kg of ECE and DK (Figure 1B). 

The total serum cholesterol levels of HFD-fed mice were significantly higher than those of NFD-fed mice. These levels were significantly decreased by the administration of 50, 100, and 150 mg/kg of ECE and DK (Figure 1C). The serum LDL levels of HFD-fed mice were significantly higher than those of NFD-fed mice. These levels were significantly decreased by the administration of 50, 100, and 150 mg/kg of ECE and DK. The 100 mg/kg of ECE contains 2.5 mg/kg of DK. Thus, we compared the effectiveness of 50 and 150 mg/kg of ECE and DK with 100 mg/kg of ECE. The LDL levels were more prominently decreased at 150 mg/kg of ECE and DK than 100 mg/kg of ECE. (Figure 1D). The serum HDL levels of HFD-fed mice were significantly lower than those of NFD-fed mice. These levels were significantly decreased by the administration of 100 and 150 mg/kg of ECE and DK (Figure 1E).

The steatosis scores in the livers of HFD-fed mice were significantly higher than those in the livers of NFD-fed mice. That score was decreased by 50, 100, and 150 mg/kg of ECE and DK. The decreasing effect was most prominent at 150 mg/kg of ECE (Figure 1F,G).

The lobular inflammation scores of HFD-fed mice were significantly higher than those of NFD-fed mice. These scores were decreased by 50, 100, and 150 mg/kg of ECE and DK. The decreasing effect was most prominent at 150 mg/kg of ECE (Figure 1F,H).

The ballooning scores of HFD-fed mice were significantly higher than those of NFD-fed mice (Figure 1F,J). These scores were decreased by 50, 100, and 150 mg/kg of ECE and DK. The decreasing effect did not differ significantly between 100 and 150 mg/kg of ECE (Figure 1F,J)

The steatosis area evaluated by oil red O intensity was significantly increased by HFD. It was significantly decreased by 50, 100, and 150 mg/kg of ECE and DK. The decreasing effect was most prominent at 150 mg/kg of ECE (Figure 1I,K).

### 2.2. ECE and DK Led to Decreased M1 Macrophages, Increased M2 Macrophages, and Decreased IL-6 and TNF-α in the Lymphatic Vasculature of Liver

**Figure 2:**
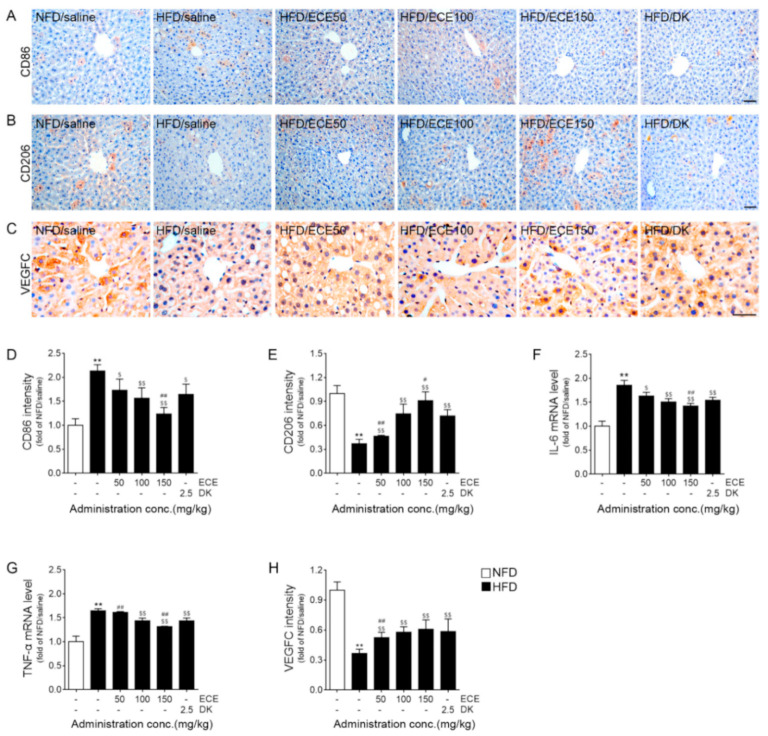
Regulatory effects of ECE and DK on M1, M2, and inflammation-related factors around the lymphatic vessels of HFD-fed mice. (**A**,**D**) The M1 (CD86) expression level around the lymphatic vessels of the liver was increased by the HFD/saline and decreased by HFD/ECE or DK treatment. (**B**,**E**) The M2 (CD206) expression level around the lymphatic vessels of the liver was decreased by the HFD/saline and increased by HFD/ECE or DK treatment. The graph of the histological images was quantified with positive signals around the lymphatic vessels of liver tissue. (**F**,**G**) The mRNA levels of IL-6 (**F**) and TNF-α (**G**) were increased by the HFD/saline groups and decreased, by HFD/ECE or DK treatment, respectively. (**C**,**H**) The VEGFC expression level around the lymphatic vessels of the liver was decreased by the HFD/saline and increased by HFD/ECE or DK treatment. Scale bar = 100 μm. Data are mean ± SD. ** *p* < 0.01 vs. NFD/saline; $ *p* < 0.05 and $$ *p* < 0.01 vs. HFD/saline; # *p* < 0.05 and ## *p* < 0.01 vs. HFD/ECE100 (Mann–Whitney U test). CD86, cluster of differentiation 86; CD 206, cluster of differentiation 206; DK, dieckol; ECE, *Ecklonia cava* extract; HFD, high-fat diet; IL-6, interleukin 6; NFD, normal-fat diet; TNF-α, tumor necrosis factor-α; VEGFC, vascular endothelial growth factor C.

The expression of CD86 (a marker of the M1 phenotype) around the lymphatic vessels of the livers of HFD-fed mice was significantly higher than that in the livers of NFD-fed mice. It was significantly decreased by the administration of 50, 100, and 150 mg/kg of ECE and DK. The decreasing effect was most prominent at 150 mg/kg of ECE (Figure 2A,D). The expression of CD206 (a marker of the M2 phenotype) around the lymphatic vessels of the livers of HFD-fed mice was significantly lower than that in NFD-fed mice. It was significantly increased by the administration of 50, 100, and 150 mg/kg of ECE and DK. The increasing effect was most prominent at 150 mg/kg of ECE (Figure 2B,E).

The expression of IL-6 in the livers of HFD-fed mice was significantly higher than that in the livers of NFD-fed mice. This expression was significantly decreased by the administration of 50, 100, and 150 mg/kg of ECE and DK. The most prominent decreasing effect was shown at 150 mg of ECE (Figure 2F). The expression of TNF-α in the livers of HFD-fed mice was significantly higher than that in the livers of NFD-fed mice. This expression was significantly decreased by the administration of 100 and 150 mg/kg of ECE and DK. The most prominent decreasing effect was shown at 150 mg of ECE (Figure 2G). 

The expression of VEGFC around the lymphatic vessels of livers of HFD-fed mice was significantly lower than that in the livers of NFD-fed mice. This expression was significantly increased by the administration of 50, 100, and 150 mg/kg of ECE and DK. There was no statistically significant difference in the increasing effect between 100 and 150 mg/kg of ECE and DK (Figure 2C,H).

### 2.3. ECE and DK Increased the Expression of VEGFR3/pERK or VEGFR3/PI3K/pAKT around the Lymphatic Vessels of Liver

**Figure 3:**
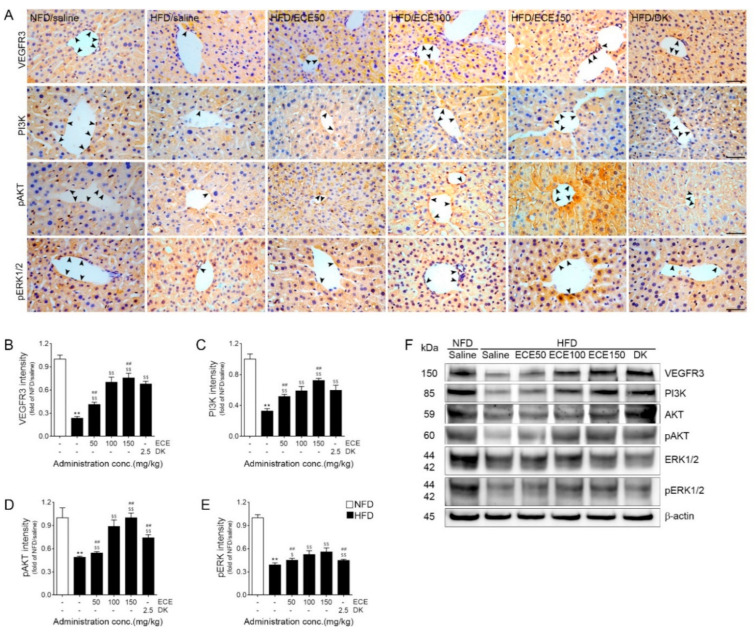
Regulatory effects of ECE and DK on the VEGFR3 pathway in the lymphatic vasculature of liver tissue of HFD-fed mice. (**A**–**E**) The levels of VEGFR3 (**A**, first line and **B**), PI3K (**A**, second line and **C**), pAKT (**A**, third line and **D**), and pERK (**A**, fourth line and **E**) in the lymphatic vasculature of liver were decreased by HFD/saline and increased after treatment with ECE or DK. The graph of the histological images was quantified with positive signals (black arrows) around the lymphatic vessels of liver tissue. Scale bar = 100 μm. (**F**) The immunoblotting results show expression of VEGER3, PI3K, AKT, pAKT, ERK1/2, and pERK1/2. Data are mean ± SD. ** *p* < 0.01 vs. NFD/saline; $ *p* < 0.05 and $$ *p* < 0.01 vs. HFD/saline; ## *p* < 0.01 vs. HFD/ECE100 (Mann–Whitney U test). DK, dieckol; ECE, *Ecklonia cava* extract; HFD, high-fat diet; NFD, normal-fat diet; PI3K, phosphoinositide 3-kinases; pAKT, phosphorylated protein kinase B; pERK1/2, phosphorylated extracellular signal-regulated kinases1/2; VEGFR3, vascular endothelial growth factor receptor 3.

The expression levels of VEGFR3, PI3K, and pAKT around the lymphatic vessels of the livers of HFD-fed mice were significantly lower than those in the livers of NFD-fed mice. These expression levels were significantly increased by the administration of 50, 100, and 150 mg/kg of ECE and DK. The most prominent increasing effect was shown when 150 mg/kg of ECE was administered (Figure 3A–D,F and Figure S2A–C).

The expression of pERK in the lymphatic vasculature of the livers of HFD-fed mice was significantly lower than that in the livers of NFD-fed mice. That expression was significantly increased by the administration of 50, 100, and 150 mg/kg of ECE and DK. The most prominent increasing effect was shown when 100 and 150 mg/kg of ECE were administered (Figure 3A,E,F and Figure S2D).

### 2.4. ECE and DK Increased the Expression of LYVE-1 and Decreased VE-Cadherin in the Lymphatic Vasculature of Liver

**Figure 4:**
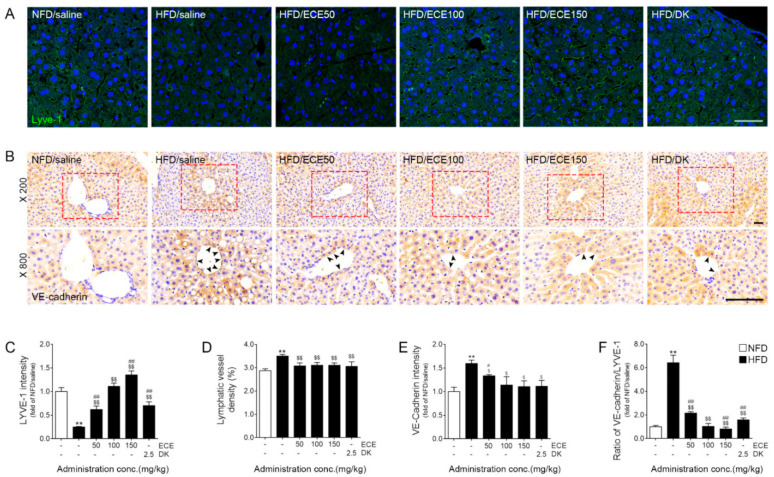
Effects of ECE and DK on LYVE-1 and VE-cadherin modulation in the lymphatic vasculature of liver tissue in HFD-fed mice. In the lymphatic vasculature of liver tissue, (**A**,**C**) the LYVE-1 expression levels were decreased by HFD/saline. The addition of ECE and DK increased the LYVE-1 expression levels. (**D**) The lymphatic vessel density (area of the lymphatic vessels divided by area of the defined tissue area) was increased HFD/saline and decreased ECE or DK treatment. (**B**,**E**) VE-cadherin protein levels were increased by HFD/saline and decreased by ECE or DK treatment. (**F**) The VE-cadherin/LYVE-1 ratio was increased by HFD/saline and decreased by ECE or DK treatment. The graph of the histological images was quantified with positive signals (black arrows) around the lymphatic vessels of liver tissue. Scale bar = 100 μm. Data are mean ± SD. ** *p* < 0.01 vs. NFD/saline; $ *p* < 0.05 and $$ *p* < 0.01 vs. HFD/saline; # *p* < 0.05 and ## *p* < 0.01 vs. HFD/ECE100 (Mann–Whitney U test). DK, dieckol; ECE, *Ecklonia cava* extract; HFD, high-fat diet; LYVE-1, lymphatic vessel endothelial hyaluronan receptor-1; NFD, normal-fat diet; VE-cadherin, vascular endothelial-cadherin.

The expression of LYVE-1 in the lymphatic vasculature of the livers of HFD-fed mice was significantly lower than that in the livers of NFD-fed mice. This expression was significantly increased by the administration of 50, 100, and 150 mg/kg of ECE and DK. The increasing effect was most prominent at 150 mg/kg of ECE (Figure 4A,C). The lymphatic vessel density of the livers of HFD-fed mice was significantly higher than NFD-fed mice. This expression was significantly decreased by the administration of 50, 100, and 150 mg/kg of ECE and DK (Figure 4A,D).

The expression of VE-cadherin around the lymphatic vessels of the livers of HFD-fed mice was significantly higher than NFD-fed mice. That expression was decreased by 50, 100, and 150 mg/kg of ECE and DK. The decreasing effect did not differ significantly between 100 and 150 mg/kg of ECE and DK (Figure 4B,E).

The ratio of the expression of VE-cadherin and LYVE-1 in the lymphatic vasculature of the livers of HFD-fed mice was significantly higher than that in the livers of NFD-fed mice. This ratio was decreased by 50, 100, and 150 mg/kg of ECE and DK. The decreasing effect was most prominent at 150 mg/kg of ECE (Figure 4F). 

## 3. Discussion

The number of NAFLD patients in the United States will increase to an estimated 100.9 million by 2030 [46]. The incidence of NAFLD is increasing in parallel to metabolic syndrome, which is a worldwide pandemic [47]. Many studies have demonstrated that the main pathogenic mechanism involved in the progression of NAFLD is the inflammatory response that is mediated by macrophages, which is the main component of innate immunity [48]. Metabolic inflammation is distinct from the acute inflammation induced by pathogens such as bacteria [49]. Acute inflammation shows a strong immune reaction immediately after exposure to bacteria, and it rapidly disappears, followed by the removal of the pathogen [49]. However, metabolic inflammation has the feature of persistent, low-grade, sterile inflammation [49]. By stimulating factors, macrophages change their phenotype into M1, which is the inflammation-promoting type, or M2, which is the inflammation-suppressive type [50]. Increased inflammatory cytokines such as IL-6 and TNF-α are also involved in the progression of NAFLD [51].

Here, food intake and change of body weights was increased by HFD, and it was significantly decreased by the administration of ECE or DK (Figure 1A,B). The serum levels of total cholesterol and LDL were also increased by HFD and were attenuated by the administration of ECE or DK (Figure 1C,D). Previously, it was reported that ECE leads to decreased body weight gain and food intake, which are increased by HFD [41]. It is well known that dietary interventions, such as low-calorie diet, are necessary for treating NAFLD [52]. Moreover, it is known that weight loss also shows an effect on decreasing NAFLD in humans [53]. Thus, it seems that decreasing weight gain and food intake by administration of ECE or DK leads to decreased NAFLD disease activity in the HFD-fed animal. 

Isolated steatosis, the most benign form of NAFLD, is characterized by the accumulation of macrovesicular lipid in 5% or more hepatocytes [54]. In NASH, hepatocellular ballooning, Mallory–Denk bodies, and inflammation are demonstrated [54]. Chronic inflammation is associated with fibrosis, and it might progress to cirrhosis [54]. NAFLD disease activity is usually evaluated by scoring steatosis, ballooning, and lobular inflammation [54].

Here, the scores of steatosis, lobular inflammation, and ballooning were increased by HFD, and they were decreased by the administration of ECE or DK (Figure 1F–K).

The infiltration of the M1 phenotype in the lymphatic vasculature of liver was increased by HFD, but M2 was decreased. Through the administration of DK or ECE, the infiltration of M1 was decreased and that of M2 was increased. The expression of IL-6 and TNF-α in the liver was increased by HFD, and it was decreased by the administration of ECE or DK (Figure 2A,B,D–G).

It is well known that lymphangiogenesis is induced by chronic and acute inflammation [55]. Inflammation results in structural changes and dysfunction of lymphatic vessels. Thus, changes in lymphatic vessels affect the modulation of inflammation and adaptive immune responses [55,56,57]. Lymphatic vessels are also involved in the modulation of metabolic inflammation, which is associated with metabolic diseases such as obesity. The inactivation of PROX-1 in mice results in changes in the structure of lymphatic vessels, which are leakier and lead to obesity in adulthood [58]. The adipose tissue of obese subjects showed decreased expression of VEGF-C compared to lean control subjects [59]. The interruption of aortic lymph flow induced by lymphatic ligation leads to inflammation of the adventitia and progression of atherosclerotic plaques in mice with hypercholesterolemia. This suggests that decreased clearance of atherogenic factors through lymphatic drainage leads to the formation of atheroma in the arterial wall [60]. Because lymphatic vessels are involved in the clearance of pathogens, lipids, and immune cells, lymphangiogenesis may contribute positively to resolving inflammation by increasing lymph efflux or drainage of lymphatic fluid. However, several studies have demonstrated that inflammation-induced lymphangiogenesis is not always advantageous, because newly made lymphatic vessels are dysfunctional [61,62].

Previous studies have demonstrated that lymphatic vessels were expanded in liver cirrhosis and NASH [15,37]. In a NASH animal model, which was created by HFD, lymphatic expansion was also observed [37]. Even though the HFD-induced increased lymphatic vessel density, and lymphatic lineage-specific genes such as PROX-1, LYVE-1, and VEGFR-3 were decreased [37]. Those genes were also decreased by cholesterol, and downregulation of those genes was related with the impairment of the structural stability of lymphatic vessels [15]. Decreased PROX-1, LYVE-1, and VEGFR-3 were also associated with decreased lymphatic drainage, which was evaluated by FITC-dextran in animal models of NASH [37]. The size of 500-kD FITC-dextran is similar to that of LDL, which is normally transported by the lymphatic vessels [63]. After the injection of FITC-dextran in the liver parenchyma, the amount of FITC-dextran in the liver-draining portal lymph nodes and that in the non-liver draining inguinal lymph nodes were compared [37]. By HFD, the amount of FITC-dextran in the portal draining lymph nodes was decreased compared to that in non-liver draining lymph nodes [37]. The expression of VE-cadherin was increased, accompanied by decreased lymphatic drainage [37]. It is suggested that the dedifferentiation of LyECs, which is confirmed by decreasing lymphatic lineage-specific genes, was associated with decreasing lymphatic drainage in HFD-induced NASH [37]. The expanded lymphatic vasculature in NASH becomes less permeable [38]. The immune cells or macromolecules are transported through permeable button-like junctions. Thus, the modulation of permeability is essential to lymphatic drainage. The authors also showed that the injection of recombinant VEGF-C restored lymphatic drainage [37]. By the administration of recombinant VEGF-C, lymphangiogenesis also increased [37]. It seems that functional lymphatic vessel formation is more important in mitigating inflammation in the liver. Increased lymphangiogenesis, without an improvement in lymphatic drainage or lymphatic vessels that consist of differentiated cells, could not contribute to the resolution of inflammation in the liver.

Here, the expression of VEGFC and VEGFR3 was decreased by HFD, and it was restored by the administration of ECE or DK. The expression of the PI3K, pAKT, and pERK signaling pathways, which are associated with lymphangiogenesis, was decreased in HFD-fed mice and was increased by the administration of ECE or DK (Figure 2C,H, Figure 3 and Figure S2). The expression of LYVE-1 was decreased in HFD-fed mice, and it was increased by the administration of ECE or DK. The lymphatic vessel density was increased by HFD, and it was decreased by ECE or DK. Interestingly, the expression of VE-cadherin was increased by HFD, and it was decreased by the administration of ECE or DK (Figure 4). It seemed that DK and ECE increased lymphangiogenesis and inhibited decreasing permeability through HFD.

A previous study showed that dieckol-enriched extracts from Laminaria japonica attenuated NAFLD induced by HFD by modulating hepatic lipid metabolism and increasing hepatic fatty acid β-oxidation [45]. Other studies have demonstrated that ECE decreased inflammatory cytokines such as TNF-α or IL-1β and induced decreasing hepatic lipid synthesis, thus attenuating NAFLD [64]. However, so far, there is no study demonstrating the effect of ECE or DK on lymphatic function modulation in the liver for attenuating NAFLD.

Here, our results showed that ECE or DK decreased inflammatory cytokines, such as IL-6 and TNF-α, and M1 macrophages in the livers of HFD-fed mice. In addition, ECE or DK induced lymphangiogenesis by upregulating VEGFR3/pERK or VEGFR3/PI3K/pAKT and inhibited change in the permeability of lymphatic vessels, which was evaluated by the expression of VE-cadherin. Moreover, ECE or DK decreased weight gain and food intake, which have been known as essential interventions for treating NAFLD in humans. By modulating lymphangiogenesis, modulating lymphatic permeability, and decreasing food intake, ECE or DK attenuated NAFLD.

## 4. Materials and Methods 

### 4.1. The HFD-Induced NAFLD Mouse Model and Material Preparation

Seven-week-old C57BL/6N male mice were purchased from Orient Bio (Sungnam, Korea) and allowed to adapt for 1 week. The animals were maintained in cages in a room with controlled conditions (temperature of 23 °C with 50% humidity under a 12 h light/12 h dark cycle).

At 8 weeks of age, the mice were randomly categorized into 6 groups. Based on other studies [65] in which HFD-induced animals were used as NAFLD animal models, they were divided into 6 groups and fed for 8 weeks as follows: (i) NFD/saline (normal-fat diet for 4 weeks + co-administered with 0.9% normal saline for 4 weeks by oral administration), (ii) HFD/saline (high-fat diet for 4 weeks + co-administered with 0.9% normal saline for 4 weeks by oral administration), (iii) HFD/ECE 50 (high-fat diet for 4 weeks + administered with ECE 50 mg/kg/day for 4 weeks by oral administration), (iv) HFD/ECE 100 (high-fat diet for 4 weeks + co-administered with ECE 100 mg/kg/day for 4 weeks by oral administration), (v) HFD/ECE 150 (high-fat diet for 4 weeks + co-administered with ECE 150 mg/kg/day for 4 weeks by oral administration), and (vi) HFD/DK (high-fat diet for 4 weeks + co-administered with DK 2.5 mg/kg/day for 4 weeks by oral administration). The ECE and DK used in this study were isolated as described in a previous study [44].

After 8 weeks, the serum and liver tissue samples were collected in accordance with ethical principles issued by the Institutional Animal Care and Use Committee of Gachon University, which approved the study (approval no. LCDI-2019-0130).

### 4.2. Serum Preparation and Total Cholesterol, LDL, and HDL Measurements

For serum preparation, blood was collected in serum separation tubes (BD Microtainer^®^ blood collection tube, Becton, Dickinson and Company, Franklin Lakes, NJ, USA). The collected blood was incubated at room temperature for 20 min. After 20 min, blood was centrifuged at 2000 rpm for 20 min at room temperature. Thereafter, the supernatant (serum) was aliquoted to another new e-tube. The obtained serum was used to carry out clinical chemistry tests (KPNT, Gyunggi-do, Korea). The selected analyses were the total serum cholesterol, LDL, and HDL levels.

### 4.3. Hematoxylin and Eosin Staining for NAFLD Activity Measurement

Liver paraffin tissue slides (7 µm) were deparaffinized and rehydrated. The deparaffinized slides were stained with hematoxylin (DAKO, Glostrup, Denmark) and eosin (Sigma-Aldrich, St. Louis, MO, USA) according to the manufacturer’s instructions. The stained sections were visualized by light microscopy (Olympus Optical Co., Tokyo, Japan) and morphological changes were analyzed. NAFLD activity examination was performed in a blinded fashion using the following criteria [52,65,66]. The NAFLD activity score was measured using the following individual scores: (i) steatosis (0, <5%; 1, <33%; 2, <66%; 3, >66%), (ii) lobular inflammation (0, none; 1, <2 foci/200× field; 2, <4 foci/200× field; 3, >4 foci/200× field), and (iii) ballooning (0, none; 1, few balloon cells; 2, prominent ballooning).

### 4.4. Oil Red O Staining for Hepatic Lipid Accumulation Measurement

Liver frozen tissue slides (10 µm) were stained with oil red O (Sigma-Aldrich, St. Louis, MO, USA) to identify lipid accumulation in hepatocytes due to lymphangiogenesis. Liver frozen tissue slides were washed with distilled water. After air-drying, the slides were loaded in 1,2-propanediol (Sigma-Aldrich) for 5 min and stained with prewarmed oil red O solution for 10 min at 60 °C. After washing with distilled water, the slides were mounted with a coverslip using DPX mounting medium (Sigma-Aldrich). The oil red O-stained images were visualized by light microscopy (Olympus Optical), and quantification of the intensity of the red color was measured using ImageJ software (National Institutes of Health).

### 4.5. 3,3′-Diaminobenzidine (DAB) Staining 

The paraffinized liver tissue slides (7 µm) were deparaffinized and rehydrated. The deparaffinized liver tissue slides were incubated in normal animal serum to block a nonspecific background, then applied with primary antibodies (Table S2) at 4 °C. After wash slides, the probed slides were soaked with biotinylated secondary antibodies from the ABC kit (Vector Laboratories, Burlingame, CA, USA) for 1 h at room temperature in normal animal serum. After washing with PBS, the antibody-attached slides were developed with a DAB substrate for 5–15 min and were stained with hematoxylin to confirm the tissue nucleus. Finally, the stained slides were mounted with a coverslip using DPX mounting medium (Sigma-Aldrich, St. Louis, MO, USA). The developed DAB images were visualized by light microscopy (Olympus Optical Co., Tokyo, Japan), and quantification of DAB intensity was measured, only for the positive signal around the lymphatic vessels of the liver tissue, using ImageJ software (National Institutes of Health, Bethesda, MD, USA).

### 4.6. Isolation of RNA and Quantitative Real-Time-Polymerase Chain Reaction (qRT-PCR)

Fifty milligrams of frozen liver tissues were ground using liquid nitrogen and then homogenized in 500 µL of RNiso (Takara, Shiga, Japan). Homogenates were mixed with 100 µL of chloroform and centrifuged at 12,000× *g* for 15 min at 4 °C. The aqueous layers were collected in cleaned tubes, mixed with 250 µL of isopropanol, and centrifuged at 12,000× *g* for 15 min at 4 °C. Isolated RNA samples were washed with 500 µL of 75% ethanol and dissolved in 30 µL of diethyl pyrocarbonate-treated water. For qRT-PCR, RNA was converted to cDNA using a PrimeScript First Strand cDNA Synthesis Kit (Takara, Shiga, Japan). After synthesis, qRT-PCR was performed using the CFX 384 Touch™ Real-Time PCR detection system (Bio-Rad Laboratories, Irvine, CA, USA). The reaction efficiency and cycle threshold numbers were determined using CFX Manager™ software (Bio-Rad Laboratories, Hercules, CA, USA). For internal control, *actb* was used, and the primer sequences for the target genes are detailed in Table S1.

### 4.7. Immunofluorescence

Antigen retrieval was performed on the deparaffinized liver tissue slides (7 µm) using sodium citrate buffer (pH 6.0), and the slides were blocked in normal animal serum. After washing the slides, LYVE-1 antibody (Table S2) was loaded onto the slides for 2 h at room temperature, and it was left at 4 °C overnight. Then, the probed slides were soaked with fluorescence-conjugated secondary antibody Alexa Fluor 488 (Invitrogen, Waltham, MA, USA) for 1 h at room temperature in normal animal serum. After washing with PBS containing 0.1% Tween-20 (TW2001, LPS solution, Daejeon, Korea), the slides were incubated with 4′,6-diamidino-2-phenylindole (Sigma-Aldrich, St. Louis, MO, USA) solution for 30 s, rinsed with PBS, and mounted with vector shield solution (Vector Laboratories, Burlingame, CA, USA). The fluorescence signal was detected using a confocal microscope (LSM 710, Carl Zeiss, Oberkochen, Germany).

### 4.8. Isolation of Protein and Western Blotting

Eighty milligrams of frozen liver tissues were homogenized in 500 µL of RIPA buffer (EzRIPA, ATTO, Japan) containing proteinase and phosphatase inhibitors. The homogenized liver tissues were sonicated and centrifuged at 14,000× *g* for 15 min at 4 °C. The supernatants were transferred to cleaned tubes. The isolated proteins were quantified using a bicinchoninic acid assay kit (BCA kit; Thermo Fisher Scientific, Inc., Waltham, MA, USA). 

Equal amounts of proteins were separated by 10 or 12% sodium dodecyl sulfate polyacrylamide gel electrophoresis. The proteins were transferred to a polyvinylidene fluoride membrane using a power station (WSE-3500, ATTO, Osaka, Japan). Then, the membranes were incubated with 5% skim milk (SKI500, LPS solution, Daejeon, Korea) in Tris-buffered saline containing 0.1% Tween-20 (TTBS). After washing with TTBS, the membranes were incubated with diluted primary antibodies (as listed in Table S2). After washing three times with TTBS, the membranes were incubated with secondary antibodies. Then, the membranes were developed by chemiluminescence using LAS-4000s (GE Healthcare, Chicago, IL, USA). 

### 4.9. Statistical Analysis

Nonparametric tests were used in this study. The Kruskal–Wallis test was used to determine the significance of differences between the six groups. If a significant difference was confirmed by Kruskal–Wallis, multiple comparisons were performed using Mann–Whitney U test. Experiments were performed in triplicate for each animal, and the results are presented as the mean ± standard deviation (SD). Statistical analysis was conducted using SPSS version 22 (IBM Co., Armonk, NY, USA).

* indicates compared with NFD/saline.

$ indicates compared with HFD/saline.

# indicates compared with HFD/ECE100.

## Data Availability

All data supporting the conclusions of this article are included in this article.

## Supplementary Materials

The following are available online at https://www.mdpi.com/article/10.3390/md19090495/s1: Figure S1: The chemical structure of compound dieckol (DK) of *Ecklonia cava*, Figure S2: Regulatory effects of ECE and DK on the VEGFR3 pathway in the liver tissue of HFD-fed mice, Table S1: List of primers for qRT-PCR and Table S2: List of antibodies for staining (immunohistochemistry and immunofluorescence) and Western blotting.
