# Supplementary material for: Dieckol Decreases Caloric Intake and Attenuates Nonalcoholic Fatty Liver Disease and Hepatic Lymphatic Vessel Dysfunction in High-Fat-Diet-Fed Mice
Source: Mar Drugs. 2021 Aug 30;19(9):495. doi: 10.3390/md19090495 (PMC8469311; doi:10.3390/md19090495)
Supplement: Supplementary file 1 [file marinedrugs-19-00495-s001.zip › marinedrugs-1327132-supplementary.pdf]

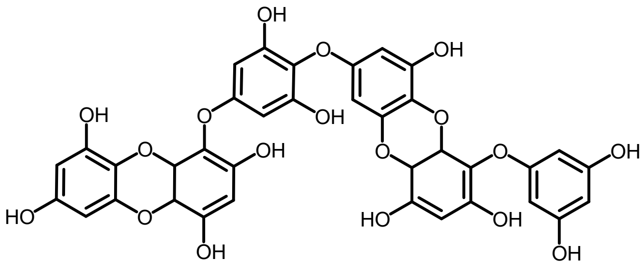

Dieckol (DK)

Figure S1. The chemical structure of compound dieckol (DK) from *Ecklonia cava.*

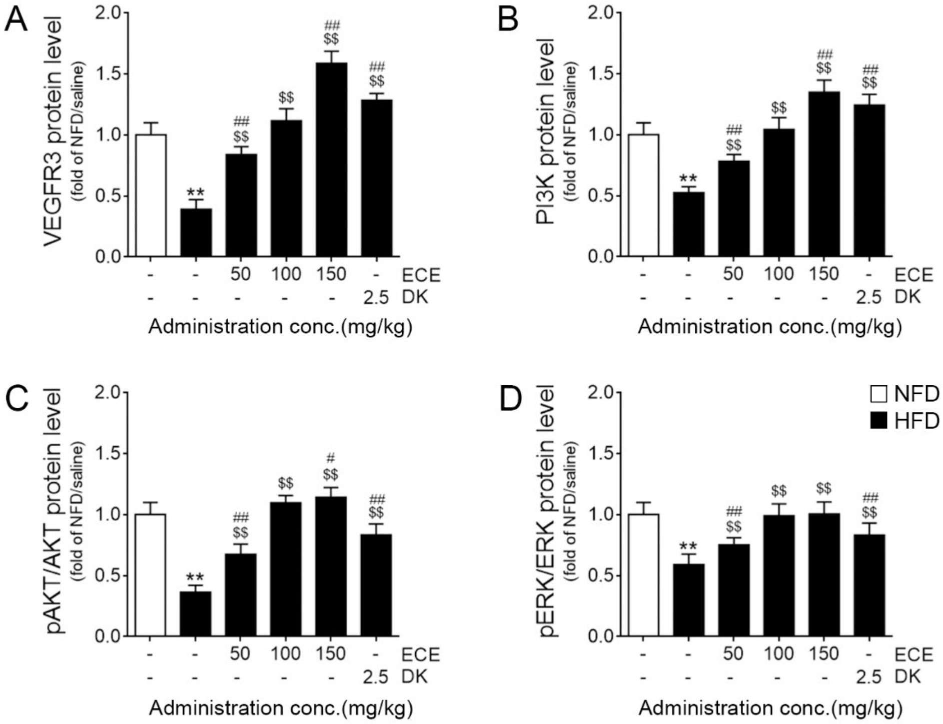

Figure S2. Regulatory effects of ECE and DK on the VEGFR3 pathway in the liver tissue of HFD-fed mice. (A–D) The levels of VEGFR3 (A), PI3K (B), pAKT/AKT (C), and pERK/ERK (D) in the liver were decreased by HFD/saline and increased after treatment with ECE or DK. The graph of the immunoblotting was quantified in liver tissue. Data are mean ± SD. **, $p < 0.01$ vs. NFD/saline; $$, $p <$ 0.01, vs. HFD/saline; #, *p,* < 0.05 and ##, *p*, < 0.01 vs. HFD/ECE100 (Mann-Whitney U test). DK, dieckol; ECE, *Ecklonia cava* extract; HFD, high-fat diet; NFD, normal fat diet; PI3K, phosphoinositide 3-kinases; pAKT, phosphorylated protein kinase B; pERK1/2, phosphorylated extracellular signal-regulated kinases1/2; VEGFR3, vascular endothelial growth factor receptor 3.

Table S1. List of primers for qRT-PCR

Gene		Primers
Actb	Forward	5'-CCGTAAAGACCTCTATGCCAAC-3′
	Reverse	5'-GCAGTAATCTCCTTCTGCATCC-3′
IL-6	Forward	5'-TCCGGAGAGGAGACTTCACA-3′
	Reverse	5'-CATAACGCACTAGGTTTGCCG-3′
TNF-α	Forward	5'-TGTCTACTCCTCAGAGCCCC -3′
	Reverse	5'-GACCCGTAGGGCGATTACAG-3′

Table S2. List of antibodies for staining (immunohistochemistry and immunofluorescence) and western blotting

Antigen	Host	Company	Catalog no.	Dilution rate	
				staining	Western bolotting
CD86	Mouse	Santa cruz biotechnology	sc-19617	1:100	-
CD206	Mouse	Santa cruz biotechnology	sc-58987	1:100	-
VEGFC	Rabbit	Invitrogen	PA5-29772	1:100	-
VEGFR3	Mouse	Santa cruz biotechnology	sc-514825	1:100	1:100
PI3K	Mouse	Santa cruz biotechnology	sc-376112	1:50	1:500
AKT	Mouse	BD	BD610860	-	1:500
pAKT	Mouse	Santa cruz biotechnology	sc-514032	1:50	-
pAKT	Rabbit	Cell signaling	4060	-	1:1000
ERK1/2	Rabbit	Cell signaling	9102	-	1:1000
pERK1/2	Rabbit	Invitrogen	PA5-37824	1:100	1:500
β-actin	Rabbit	Cell signaling	4967	-	1:500
LYVE-1	Rabbit	LSBio	LS-B10511	1:100	-
VE-cadherin	goat	Santa cruz biotechnology	sc-6458	1:100	-
